# High-Risk Human Papillomavirus Oncogenic E6/E7 mRNAs Splicing Regulation

**DOI:** 10.3389/fcimb.2022.929666

**Published:** 2022-06-27

**Authors:** Yunji Zheng, Xue Li, Yisheng Jiao, Chengjun Wu

**Affiliations:** ^1^ School of Pharmacy, Binzhou Medical University, Yantai, China; ^2^ School of Biomedical Engineering, Dalian University of Technology, Dalian, China

**Keywords:** high-risk HPVs, cervical cancer, HNSCC, E6/E7, splicing, splicing factors

## Abstract

High-risk human papillomavirus infection may develop into a persistent infection that is highly related to the progression of various cancers, including cervical cancer and head and neck squamous cell carcinomas. The most common high-risk subtypes are HPV16 and HPV18. The oncogenic viral proteins expressed by high-risk HPVs E6/E7 are tightly involved in cell proliferation, differentiation, and cancerous transformation since E6/E7 mRNAs are derived from the same pre-mRNA. Hence, the alternative splicing in the E6/E7-coding region affects the balance of the E6/E7 expression level. Interrupting the balance of E6 and E7 levels results in cell apoptosis. Therefore, it is crucial to understand the regulation of E6/E7 splice site selection and the interaction of splicing enhancers and silencers with cellular splicing factors. In this review, we concluded the relationship of different E6/E7 transcripts with cancer progression, the known splicing sites, and the identified cis-regulatory elements within high-risk HPV E6/E7-coding region. Finally, we also reviewed the role of various splicing factors in the regulation of high-risk HPV oncogenic E6/E7 mRNA splicing.

## Human Papillomavirus Infection and Related Cancers

Human papillomaviruses (HPV) are non-enveloped, small, double-stranded DNA viruses. The genome size of HPVs consists of 8,000 nucleotides (zur Hausen, 2002; zur Hausen, 2009). As one of the most common sexually transmitted diseases among almost all sexually active populations, a HPV-caused viral infection usually starts at the cutaneous or mucosal epithelium at the basal layer through sexual activity or a small wound. In most cases, a HPV infection is usually asymptomatic and will disappear within 2 years (Richardson et al., 2003). However, it will develop into a persistent infection in some rare cases, further developing into malignant lesions and progressing to cancer if left untreated (Parkin and Bray, 2006; Zheng and Baker, 2006). Depending on the association of HPV subtypes with cancer progression, they are divided into high-risk HPVs and low-risk HPVs (Zheng and Baker, 2006; Stanley, 2008).

About 15% of human cancers are caused by a viral infection; a high-risk HPV infection nearly accounts for half of all cases. According to the global cancer report in 2021, about 600,000 cancer cases worldwide are reported to be associated with high-risk HPV infections, causing 340,000 deaths. Cervical cancer is one of the most prevalent cancer types tightly related to HPVs; more than 99% of cases are diagnosed with HPV infections. Moreover, recent studies have shown that anal cancer and head and neck cancer increase rapidly; about 95% of anal cancer and 80% of head and neck cancer are associated with a high-risk HPV infection (Arbyn et al., 2012). Up to now, more than 20 high-risk HPVs have been identified, among which HPV16 and HPV18 are the most popular high-risk strains (Lorincz et al., 1992; de Villiers et al., 2004; Bzhalava et al., 2013; de Villiers, 2013). Nearly half of cervical cancer cases are HPV16-positive, while HPV18 accounts for 20% of the cases (de Sanjose et al., 2010; Li et al., 2011).

High-risk HPV viral oncoproteins E6 and E7 are essential for viral replication and the termination of cell differentiation (Doorbar, 2005; Johansson and Schwartz, 2013). Furthermore, E6 and E7 play a vital role in cancer progression since they interact with cellular tumor suppressor proteins p53 and pRb. Transcription factor p53 contains a DNA binding region to activate the expression of the genes involved in DNA damage repair and cell apoptosis (Howie et al., 2009). HPV16 E6 oncoprotein interacts with p53 with the help of E6-associated protein, causing p53 degradation through the ubiquitination pathway. E6 interrupts p53 DNA binding activity, resulting in transcription inhibition by directly binding to p53. P300/CBP is a histone acetyltransferase essential for p53 production. E6 indirectly downregulates the p53 level by interacting with p300/CBP and inhibiting the p300/CBP expression level (Li and Coffino, 1996; Patel et al., 1999; Zimmermann et al., 2000). The loss of p53 may result in immortalization, followed by cancer occurrence (Scheffner et al., 1993; Martinez-Zapien et al., 2016). The tumor suppressor factor pRb plays a significant role in the regulation of cell cycle. Within the G1 phase, unphosphorylated pRb interacts with transcription factor E2F to suppress the transcription. Once pRb starts to phosphorylate gradually, the cell cycle steps into the S phase (Kato et al., 1993). HPV16 E7 interacts with pRB, causing the inactivation or degradation of pRB, thus leading to the release of E2F and thereby forcing the cell cycle to progress from G1 to the S phase (Munger et al., 2001).

The mRNAs that encode E6 and E7 are bicistronic and generated from the same pre-mRNA (Mesplede et al., 2012). Various splice sites are identified in high-risk HPV E6- and E7-coding regions. Alternative splicing decides the selection of the splice site to produce different mRNA expression variants. However, no splicing in E6 and E7 in low-risk HPVs has been reported since all the spliced E6/E7 mRNA isoforms excluded the most part of the E6-coding sequence. Hence, E6 is only translated from unspliced mRNA, and E7 is generated from several spliced mRNAs but not the unspliced mRNAs. One can speculate that the production of E7 produced from the unspliced mRNA is too low to reach the threshold that can be detected (Zheng and Baker, 2006; Ajiro and Zheng, 2014). The balance of E6/E7 level is directly controlled by alternative splicing, thereby impacting the fate of infected cells developing to apoptosis or immortalization. This review concerns the regulation of E6/E7 splicing isoforms, summarizing the identified cis-regulatory elements and the splicing factors involved in E6/E7 splicing.

## RNA Processing in General

The gaps in between the intronic region and the exonic coding regions are 5′ splice site (5′ ss) and 3′ splice site (3′ ss). The 5′ ss contains a highly conserved invariable “GU” dinucleotide, while the 3′ ss contains a conserved “AG” dinucleotide (Black, 2003; Hallegger et al., 2010). Besides the splice sites, a branch point sequence (BPS) contains a conserved adenine located at 1–40 nucleotides upstream of 3′ ss, and a polypyrimidine tract is also needed for splicing (Taggart et al., 2012). The general process of mRNA processing includes 5′ capping, polyadenylation, and mRNA splicing. 5′ capping adds a 7-methylguanosine cap to the 5′ end of the growing transcript by a 5′- to -5′ phosphate linkage (Topisirovic et al., 2011). If 5′ capping provides the ribosome with a “start-checkpoint” for translation and mRNA stabilization, the 3′ polyadenylation is an “end-check point” by adding a poly-A tail to the mRNA (Bienroth et al., 1993; Beaudoing et al., 2000). Pre-mRNA splicing is a process that brings exons together in different combinations by removing the introns to generate mature mRNA-encoding functional proteins. The occurrence of splicing requires cis-acting elements that function as splicing enhancers or silencers to interact with trans-acting factors and further constitute spliceosomes (Black, 2003; Hallegger et al., 2010). Besides RNA splicing, post-transcriptional modification is also essential to mature mRNA production, such as N6-methyl adenosine (m6A), which is the most prevalent conserved internal modification in prokaryotic and eukaryotic RNAs (Liu and Pan, 2016). However, we will only focus on the regulation of HPV oncogene E6/E7 pre-mRNA splicing in this review.

## HPV Gene Expression Is Replication Cycle Dependent

**Figure 1:**
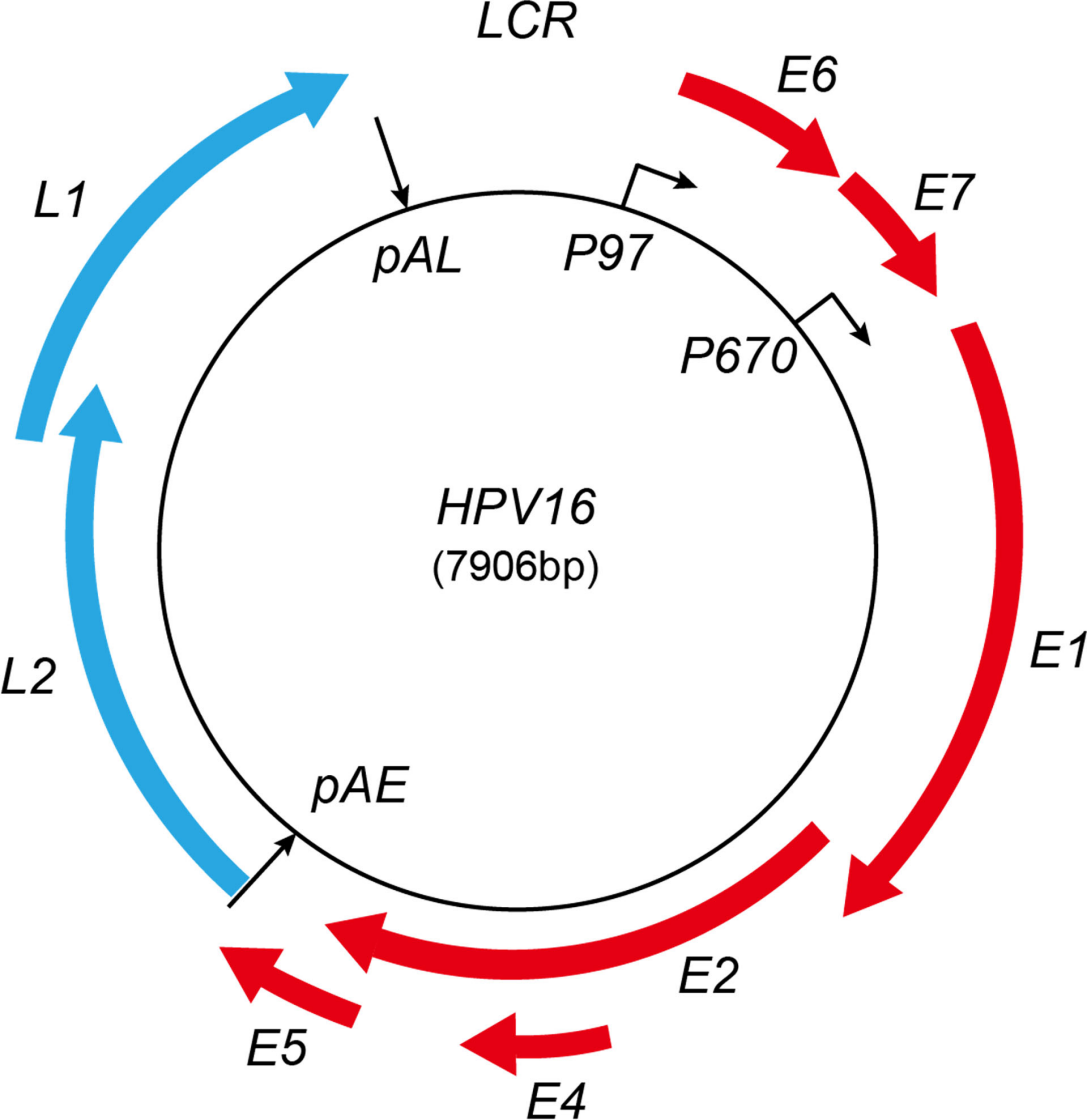
Schematic map of HPV16 genome. Red represents early proteins; blue represents late proteins. LCR, long control region; pAE, early polyadenylation signal; pAL, late polyadenylation signal. The early and late promoters are indicated. Red boxes represent viral early-protein-encoding regions; blue boxes represent late-protein-encoding regions.

Various splice sites have been identified in the HPV genome; the selection of different splice sites generates a variety of HPV mRNA transcripts to ensure that HPV expresses enough viral protein to drive their replication cycle. Therefore, the regulation of HPV gene expression is highly linked to the HPV replication cycle (Doorbar, 2005; Johansson and Schwartz, 2013). The HPV replication cycle can be divided into early and late stages. The study of the HPV replication cycle is best characterized for HPV16. In HPV16, the early promoter p97 is active during the early phase, while the late promoter p670 is active at the late phase. The HPV16 infection usually starts by gaining entry to the host cell’s nucleus in an epithelial cell located at the basal layer of the mucosal epithelium. The cellular transcription machinery helps the HPV16 genome to start replicating from early promoter p97 (Egawa, 2003; Borgogna et al., 2012; Howley and Pfister, 2015; Schafer et al., 2015). HPV16 expresses only early genes during the early stage, including E6, E7, E1, E2, E4, and E5. The HPV16 E2 protein accumulates during cell differentiation and shuts down the HPV early promoter p97. Consequently, HPV16 E6 and E7 expressions are shut off, and the HPV16 differentiation-dependent late promoter p670 is activated (Shin et al., 2007). The late promoter p670 mediates several early viral gene expressions, including E1, E2, and E4. However, when the HPV replication cycle is switched to the late stage, p670 mediates only late gene expression, including L1, L1i, and L2. All the early mRNAs produced by promoter p97 and p670 are polyadenylated at pAE, but high levels of HPV E2 protein inhibit HPV pAE, leading to the activation of HPV late L1 and L2 gene expression. The late mRNAs produced by HPV late promoter p670 are polyadenylated at pAL (Johansson and Schwartz, 2013) (**Figure 1**).

Although most of the HPV infections will be cleared by the host immune system within 2 years, some rare cases may become persistent infections and eventually progress to cancer if left untreated. It is worth mentioning that the occurrence of cancer needs to repress or mute the E2 expression since E2 plays a vital role in early gene expression (Hanahan and Weinberg, 2000). In the majority of cervical cancer cases, high-risk HPVs’ genome integrates into the host genome, and the early viral gene expression regulator E2 protein is disrupted (Pyeon et al., 2009). Within this stage, progeny virion is not produced anymore, and the overexpression of E6 and E7 is induced (Groves and Coleman, 2015). A high level and constant E6 and E7 oncoprotein expression are required for tumor formation and maintenance. However, some cervical cancer cases demonstrated that high-risk-HPV DNA keeps in an episomal form. In these cases, the differential methylation of E2 binding sites 1, 3, and 4 in long control region is related to the overexpression of the E6 and E7 proteins, further suggesting that HPV genome integration into the host genome may not be essential for malignant cellular transformation (Matsukura et al., 1989; Goodwin and DiMaio, 2000; Vinokurova et al., 2008).

## HPVs E6/E7 Splicing Isoform and Functions

**Figure 2:**
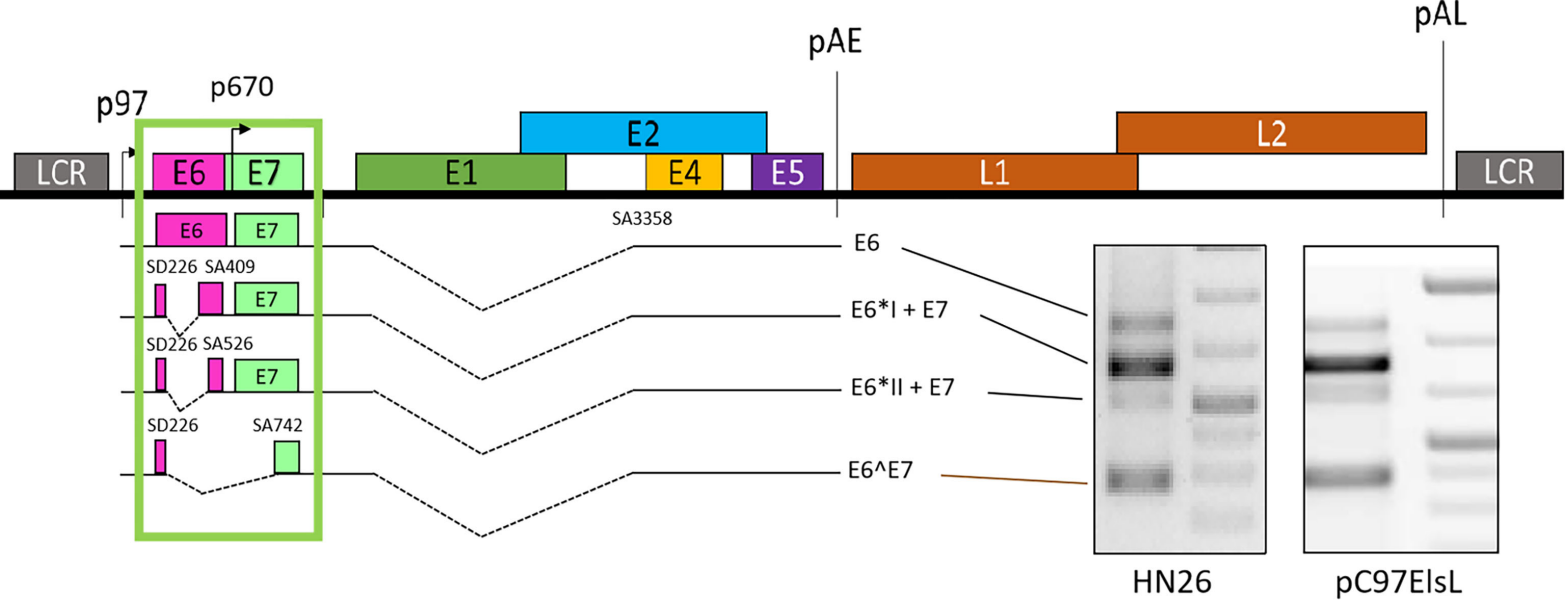
Schematic presentation of E6/E7 transcripts. The unspliced mRNA in E6 coding region generates E6 (Zheng et al., 2020), SD226^SA409 generates E6*I and E7, SD226^SA526 generates E6*II and E7, and SD226^742 generates E6^E7. All the transcripts are detected in primary head and neck cancer cell line HN26 and subgenomic plasmid pC97ElsL transfected HeLa cells.

HPV expresses multiple viral proteins to maintain the viral replication cycle. The most common high-risk subtypes HPV16 and HPV18 have been studied thoroughly. In HPV16, various splicing donors and acceptors have been characterized (**Figure 2**). The selection of different splicing donors and splicing acceptors decides the generation of viral proteins. The splicing donor SD226 alternatively spliced to SA409, SA526, and SA742 generates the E6*I, E6*II, and E6^E7 variants (**Figure 2**) (Guccione et al., 2004; Ajiro et al., 2012; Manzo-Merino et al., 2014; Olmedo-Nieva et al., 2018; Zheng et al., 2020). In HPV18, splicing donor SD233 and splicing acceptors SA416, SA3434, SA2779, and SA791 are involved in producing E6/E7 transcript variants (Vazquez-Vega et al., 2013; Cerasuolo et al., 2017; Olmedo-Nieva et al., 2018). The coding region of high-risk HPV E6/E7 contains at least one splicing donor, and an acceptor is used for alternative splicing to generate one or several isoforms termed E6*, but low-risk HPVs and beta-papillomavirus do not have such splicing sites in the E6/E7-coding region to conduct alternative splicing. We concluded the known splice sites and E6/E7 mRNA variants of high-risk HPV as those shown in **Table 1**. Since E6 and E7 are oncoproteins and play a vital role in high-risk HPV-induced cancers, the splicing mechanism of E6 and E7 is of great interest for study (Graham and Faizo, 2017; Cerasuolo et al., 2020). HPV16 oncoprotein E6 and E7 are generated from the same pre-mRNA. It is disputable that the unspliced mRNA produces an early viral E6 protein since the unspliced mRNA contains the full-length E6-encoding gene. The mRNA spliced from SD226 to SA409 produces the E6*I transcript, used for E7 production (Bernard et al., 1987). In high-risk HPV, the splicing within the E6-coding region to produce E6*I mRNA is a landmark to distinguish high- and low-risk HPV (Guccione et al., 2004; Mesplede et al., 2012; Filippova et al., 2014) replication cycle. A previous study demonstrates that the most abundant transcript isoform in HPV16-positive cervical malignant and oropharyngeal lesion is E6*I. In agreement with this observation, consistent results were identified in various cancer cell lines (Guccione et al., 2004) (**Figure 2**).

**Table 1:** Summary of identified E6/E7 splicing variants in 20 high-risk human papillomaviruses (HPVs) (Olmedo-Nieva et al., 2018).

HPV type	E6/E7 transcripts	5′ ss–3′ ss (nucleotide positions)
HPV16	E6*I	226–409 (Doorbar et al., 1990)
	E6*II	226–526 (McFarlane et al., 2015)
	E6*III	226–3,358 (Doorbar et al., 1990)
	E6*IV	226–2,709 (Schmitt et al., 2010)
	E6*V	221–409 (Ajiro et al., 2012)
	E6*VI	191–409 (Ajiro et al., 2012)
	E6^E7 (E6*X)	226–742 (Ajiro and Zheng, 2015)
	E6^E7*I	174–718 (Islam et al., 2017)
	E6^E7*II	221–850 (Islam et al., 2017)
HPV18	E6*I	233–416 (Toots et al., 2014; Ajiro et al., 2016)
	E6*II	233–3,434 (Toots et al., 2014; Ajiro et al., 2016)
	E6*III	233–2,779 (Ajiro et al., 2016)
	E6^E7	233–791 (Ajiro and Zheng, 2015; Ajiro et al., 2016)
HPV26	E6*I	173–406 (Mesplede et al., 2012)
HPV31	E6*I	210–413 (Hummel et al., 1992; Ozbun, 2002)
	E6*III	210–3,295 (Ozbun, 2002)
HPV33	E6*I	231–509 (Snijders et al., 1992)
	E6*II	231–785 (Snijders et al., 1992)
	E6*III	231–3,351 (Snijders et al., 1992)
HPV35	E6*I	228–419 (Mesplede et al., 2012)
HPV39	E6*I	231–420 (Mesplede et al., 2012)
HPV45	E6*I	230–413 (Halec et al., 2013)
HPV51	E6*I	173–406 (Mesplede et al., 2012)
HPV52	E6*I	224–502 (Halec et al., 2013)
HPV53	E6*I	236–419 (Halec et al., 2013)
HPV56	E6*I	157–420 (Mesplede et al., 2012)
HPV58	E6*I	232–510 (Li et al., 2013)
	E6*II	232–3,355 (Li et al., 2013)
HPV59	E6*I	183–582 (Halec et al., 2013)
HPV66	E6*I	157–420 (Mesplede et al., 2012)
HPV67	E6*I	224–502 (Halec et al., 2013)
HPV70	E6*I	231–422 (Halec et al., 2013)
HPV73	E6*I	227–410 (Halec et al., 2013)
HPV82	E6*I	178–411 (Mesplede et al., 2012)

The splicing between HPV16 splice site SD226 and SA409 results in the shortening of the E6-coding region. The shortened coding region produces a small protein E6*I; the E6*I open reading frame (Bernard et al., 1987) contains a weak ATG located upstream of the E7 ORF. Therefore, the ribosome may reinitiate translation from E7 ATG by leaky scanning. Zheng ZM et al. demonstrated that most E7 proteins are translated from E6*I mRNA in HPV16-positive cervical cancer cells (CaSki) and HPV18-positive HeLa cells (Schafer et al., 2015). Although increasing evidence supports this conclusion, several individual studies also presented different opinions that unspliced E6E7 RNA expresses more E7 than spliced variants. It is necessary to point out that these findings were drawn from a less stringent experimental system (Filippova et al., 2007; Manzo-Merino et al., 2014). E6*I is a multi-functional protein that has been detected in a HPV16-positive CaSki cell line and functions as an antagonist of the full-length E6 protein, further suggesting its antitumorigenic role. In HPV18, E6*I protein indirectly promotes the expression of the potential cervical cancer diagnostic marker p14ARF through p53 degradation. The overexpression of E6*I results in a slight increase of p14ARF. This result suggests that HPV18 E6*I protein may interact with p53 to prevent p53 from regulating p14ARF (Vazquez-Vega et al., 2013). Several earlier studies demonstrate that E6*I interacts with p53 to impact p53 degradation. Moreover, it has been shown that the E6*I protein does not drive keratinocyte immortalization and proliferation (Sedman et al., 1991; Pim et al., 1997). However, HPV16 produces other E6/E7 splicing isoforms, the mRNA SD226^SA526 encodes E6*II protein, while the mRNA SD226^SA742 produces E6^E7 protein. The ratio of unspliced E6, E6*I, E6*II, and E6^E7 mRNAs plays a vital role in HPV-related cancer progression. The variant E6^E7 is generally present at the lower level, while E6*I is the most abundant mRNA expressed in HPV16-infected cells or HPV16-related cancers (Ajiro et al., 2012; Ajiro et al., 2016; Olmedo-Nieva et al., 2018; Zheng et al., 2020). In addition, the splicing variant E6*II mRNA expression level is regularly higher than that of the unspliced E6 mRNA but lower than the major spliced product E6*I. However, the E6*I and E6*II mRNA levels vary in different cancers; cervical cancer samples express higher levels of E6*I and E6*II mRNAs than those in oropharyngeal cancer (Cerasuolo et al., 2017). E6*II is a suboptimal E7 producer (Schmitt et al., 2010; Schmitt and Pawlita, 2011) that has been associated with the grade of cervical lesions, but this association could not be confirmed. Very little is known about the functions of E6^E7 protein, but it has been reported to assist in the stabilization of E6, E7, and E6*I (Ajiro and Zheng, 2015). Furthermore, several suboptimal splice sites have been identified in HPV16 to generate E6/E7 mRNA variants within the E6 ORF, including SD191^SA409 (E6*VI), SD221^SA409 (E6*V), SD174^SA718 (E6^E7*I), and SD221^SA850 (E6^E7*II) (Ajiro et al., 2012; Islam et al., 2017). A previous study indicates that HPV16-positive breast cancer samples detect various E6/E7 transcript isoforms, including E6*I, E6*II, E6^E7, E6^E7*I, and E6^E7*II. This finding does not elucidate the role of these isoforms in breast cancer progression but suggests that HPV16 is transcriptionally active (Islam et al., 2017). However, the functional feature of these mRNA variants in HPV16-related cancer progression is largely unknown.

## HPV E6/E7 Splicing Regulation

The splicing regulation is tightly related to the splicing site selection regulated by the interaction of cis-acting elements and trans-acting factors. cis-acting elements provide binding motifs to interact with cellular splicing factors resulting in splicing enhancement or splicing inhibition. The cis-acting elements located in exons enhancing splicing are termed exonic splicing enhancers (ESE), while those of inhibiting splicing are termed exonic splicing silencers (ESS). Similarly, the cis-acting elements located in introns are also termed intronic splicing enhancers (Garland et al., 2007) and intronic splicing silencers (ISS). The trans-acting factors interact with cis-regulatory elements to facilitate the composition of spliceosomes, further conducting splicing from splice sites. The expression level of trans-acting factors varies in different tissues, and they may compete for binding to the cis-acting elements to promote splicing or inhibit splicing (Manley and Tacke, 1996; Wang et al., 2006; Zhou and Fu, 2013). The families of serine/arginine-rich proteins (SR proteins) and heterogeneous nuclear ribonucleoproteins (hnRNPs) are known splicing factors. The SR proteins usually function as splicing activators binding to ESE or ISE, while hnRNPs function as splicing silencers binding to ESS or ISS (Graveley et al., 1999; Hertel and Maniatis, 1999; Han et al., 2010). However, this classification is not strict; increasing evidence indicates that the role of SR and hnRNP proteins in splicing regulation may alter (Lim et al., 2011). Their opposite function in splicing depends on the positions of the splicing regulatory elements relative to the splice site. Besides SR and hnRNP proteins, various splicing factors participate in the pre-mRNA splicing process (Elliott et al., 1998).

### Cis-Regulatory Elements in E6/E7-Coding Region

The study on the mechanism of HPV splicing was well performed on the most common subtypes, including HPV16 and HPV18. Several cis-regulatory elements have been mapped, including BPS and splicing silencers. Moreover, various splicing factors have been identified to impact the production of expression of major splicing variants E6*I and E7.

**Figure 3:**
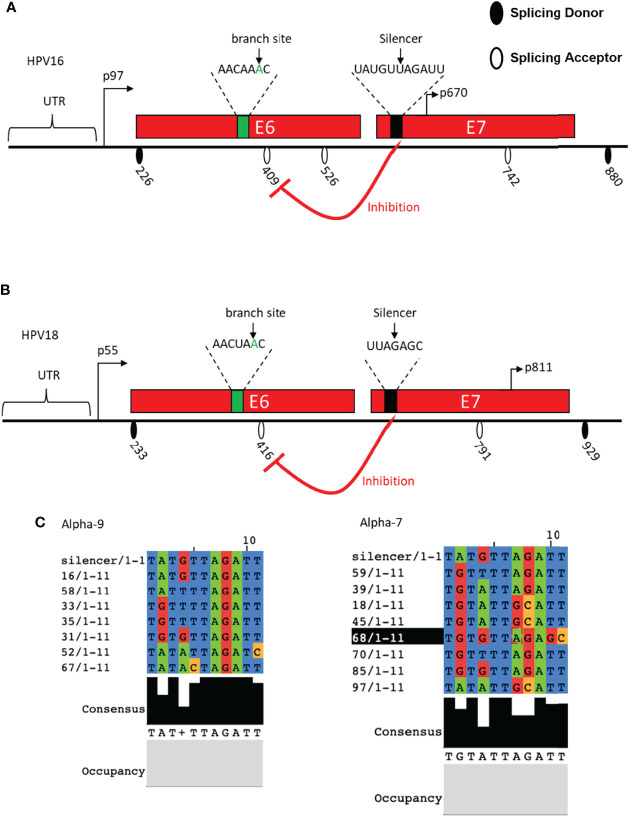
Schematic presentation of cis-regulatory elements identified in HPV16 and HPV18. **(A)** The HPV16 E6- and E7-encoding regions and the splice sites are indicated. Identified branch site and SA409 silencer are indicated. **(B)** HPV18 E6- and E7-encoding regions and the splice sites are indicated. Identified branch site and SA416 silencer are indicated. **(C)** HPV16 silencer sequence alignment with alpha-9 and alpha-7 groups.

Researchers have identified the sequence of HPV16 BPS as AACAAAC located within the E6-coding region upstream of 3′ ssSA409. The adenosine at nucleotide 385 (underlined) is the branch site to dominate the splicing to 3′ ss SA409, affecting the E7 expression. Once the point mutation was introduced to the branch site in BPS, the BPS binding activity to U2 protein was interrupted, resulting in 3′ ss SA409 splicing inhibition to 3′ ss SA409. Therefore, the pre-mRNAs contain the mutated BPS, causing inefficient splicing activity to produce very little E7 protein. Moreover, since the BPS locates in the E6-coding region, the point mutation at BPS generates the mutated E6 protein, which has little effect on p53 degradation (Ajiro et al., 2012). An earlier study identified a suboptimal BPS (AGUGAGU) on HPV16, from nucleotide 323 to 329 in the E6-coding region. The underlined guanosine at nucleotide 328 is a cryptic branch site. This cryptic BPS may play its role in keeping the E6/E7 mRNA level, but no information about E6/E7 pre-mRNA splicing pattern change has been reported (De la Rosa-Rios et al., 2006) (**Figure 3A**). In HPV18, researchers have identified two alternative branch sites for E6*I generation and E7 production. The mapped BPS is AACUAAC (from nucleotide 383 to 389); both branch sites are adenosine: one adenosine locates at nucleotide 384, and the other locates four nucleotides downstream at 388 (underlined). The selection of two alternative branch sites decides the efficiency of the E6*I splice, further affecting the production of HPV18 E6 and E7. The study has demonstrated that the E6*I splice favors the branch site at nucleotide 388 rather than the adenosine at nucleotide 384. If the preferred branch site is inactivated or mutated, cryptic splicing to 3′ ss SA636 would be activated (Brant et al., 2019) (**Figure 3B**). In general, the identified HPV18 BPS in the E6-coding region is similar to the BPS in HPV16. However, the fourth nucleotide in the BPS is differed—uridine for HPV18 and adenosine for HPV16.

In addition to BPS identification, a splicing silencer in the HPV18 E7-coding region at nucleotide 612 to 639 has been mapped to inhibit the splicing from 5′ ss SD233 to 3′ ss SA416 by binding to cellular splicing factor hnRNP A1. The interaction between the mapped silencer and hnRNP A1 inhibits HPV18 E6*I production and E7 expression. These 27 nucleotides in the length silencer contains two hnRNP A1 binding motifs: the sequence of motif one is AAGACA, and that of the other is UUAGAGC. The mutation of the motif AAGACA did not affect the SD233^SA416 splicing efficiency, but the mutation of UUAGAGC resulted in an increase in E6*I expression (Ajiro et al., 2016). This outcome suggested that the sequence UUAGAGC contributes to the interaction with hnRNP A1. Similarly, the HPV16 E7-coding region contains a splicing silencer interacting with hnRNP A1/A2 to inhibit splicing to 3′ss SA409, thereby reducing the expression of E6*I and E7 production. This silencer locates at nucleotide 594 to 604, consisting of UAUGUUAGAUU. Since HPV16 E7 genetic conservation is essential to carcinogenesis, sequence alignment was therefore performed, and it indicated that the HPV16 11 nucleotide in the length silencer is not well conserved in all high-risk HPVs (Zheng et al., 2020). However, the UAGAU is completely conserved in HPV16 belonging to the alpha-9 subgroup. In alpha-7 group, UAGAU is conserved with HPV18, HPV39, HPV59, HPV70, and HPV85 but less conserved with HPV45, HPV68, and HPV97. Interestingly, all high-risk HPVs have a “UA”-rich region close to the E7 start codon, and one can speculate that the “UA”-rich region is necessary to the silencer’s inhibitory activity, which interacted with hnRNP A1/A2 to manipulate the balancing of the E6/E7 protein level and is required for malignant transformation (Zheng et al., 2020) (**Figure 3C**).

### Splicing Factors Involved in E6/E7 mRNA Expression

**Figure 4:**
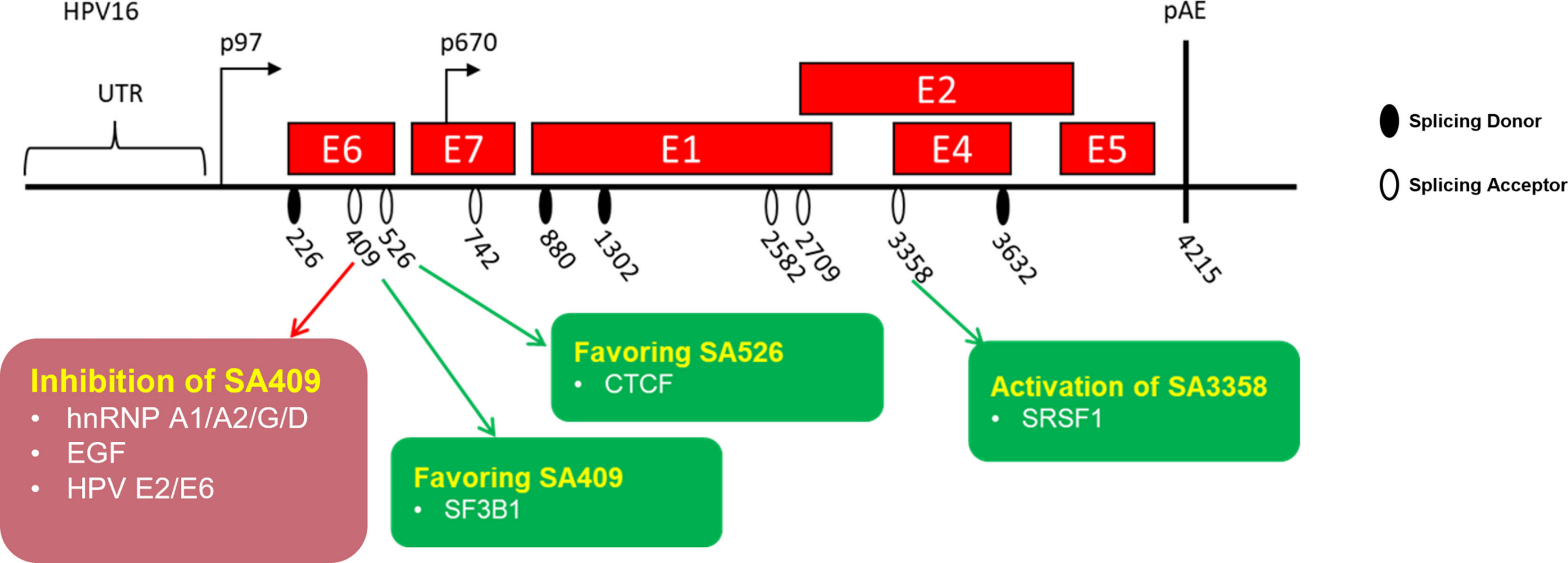
Schematic presentation of identified splicing factors involved in the regulation of human papillomaviruses early oncoprotein E6/E7 RNA splicing. HPV16-encoded viral genes are represented by a “red” box. The splice sites are indicated.

According to the effect of splicing regulation, splicing factors are classified as splicing activator and splicing repressor. However, this division is not strict; the effect of splicing factors can switch depending on the position of the splicing regulatory elements and their activity. The regulation of both HPVs E6/E7 gene expression is tightly regulated by several splicing factors, thereby affecting the splicing pattern change (**Figure 4**).

### hnRNP A1/A2/G/D

The splicing factor hnRNP A1 was identified as a splicing repressor interacting with the mapped splicing silencer (UUAGAGC) located at the HPV18 E7-coding region to inhibit splicing to 3′ ss SA416, resulting in a reduction of E6*I and E7 protein (Ajiro et al., 2016). Nevertheless, this study did not reveal how hnRNP A1 interacts with the silencer. In a recent study, the researchers reported that hnRNP A1/A2 inhibits the splicing to 3′ ss SA409 on HPV16 but that this resulted in different consequences. The overexpression of hnRNP A1 leads to an increase of unspliced E6 mRNAs at the expense of E7 mRNAs, while the overexpression of hnRNP A2 leads to alternative splicing to downstream 3′ ss SA742. The splice site SA742 is used to produce E6^E7, E1, and E4 mRNAs (Cerasuolo et al., 2017). Moreover, researchers revealed that the hnRNP A1 inhibitory effect is contributed by the interaction of hnRNP A1 C-terminus with the splicing silencer (UAGAU) in the E7-coding region (Zheng et al., 2020). Taken together, as one of the most abundant splicing factors, hnRNP A1 is reported to participate in the regulation of HPV16 early and late gene expressions, causing an E6/E7 splicing pattern change and demonstrating its vital role in the HPV replication cycle as well as malignant transformation.

A recent study demonstrates that hnRNP G inhibits splicing from HPV16 3′ ss SA409 to prevent HPV16 E7 production. The reduction of E7 levels restores the pRB levels, while the absence of hnRNP G causes a reduction of pRB. The inhibitory effect of hnRNP G may be due to the direct interaction of hnRNP G with the previously reported splicing silencer in the E7-coding region. Meanwhile, the overexpression of hnRNP G promotes the splicing from HPV16 3′ ss SA2709 to increase the production of E2 protein. The different effects of hnRNP G in the regulation of E6, E7, and E2 splicing are contributed by the different domains of hnRNP G. The hnRNP G N-terminal RGG domain and the NTD domain play an essential role in the inhibition of E6/E7 mRNA splicing, while the C-terminal region is required for E2 production. Since E2 protein shuts down the HPV16 early promoter, it thereby repressed the expression of E6 and E7 oncoproteins to pave the way for differentiation and late gene expression. The high levels of pro-apoptotic E2 protein caused by the overexpression of hnRNP G would counteract cell transformation and HPV16-related cancer progression, suggesting that hnRNP G may be used for anticancer or antiviral treatment (Hao et al., 2022).

hnRNP D has four variants, including hnRNP D37, D40, D42, and D45. All four variants have been shown to promote the intron retention of HPV16 E6 mRNAs, resulting in increasing E6 mRNA levels at the expense of E7 levels. The RRM1 and RRM2 domains of hnRNP D40 are necessary for the interaction of hnRNP D40 with HPV16 mRNAs, while the inhibitory effect of hnRNP D40 is contributed by its N-terminal region and C-terminal RGG domain. Besides splicing inhibition, hnRNP D40 promotes the intron-retained E6 mRNA expression level in the cytoplasm (Cui et al., 2022).

#### Epidermal Growth Factor

The epidermal growth factor (EGF) regulates HPV16 E6/E7 splicing via inducing the activation of the ERK1–ERK2 pathway, which inhibits splicing to 3′ ss SA409. The inhibitory effect of EGF results in the reduction of E6*I but gives rise to unspliced E6 mRNA generation (Rosenberger et al., 2010). However, the mechanism of EGF in the regulation of E6/E7 splicing remains a subject for further study.

#### 5′ Cap-Binding Factors

The previous study has reported that the splicing on 5′ capped E6/E7 mRNAs prefers the 5′ ss SD226 on HPV16. The cap-dependent HPV16 E6/E7 splicing in cervical cancer-derived cells is particularly efficient and supposed to be conducted by interacting with cap binding complex. Furthermore, the E6/E7 splicing efficiency from 5′ ss SD226 is impacted by the distance of the cap-proximal intron to the 5′ cap. Further studies identified that the optimal distance is less than 307 nucleotides, which promotes the association of 5′ splice site with U1 snRNP. Once the distance of the 5′ cap to the SD226 increases, splicing is inhibited, promoting unspliced E6 mRNA expression and decreasing E6*I production (Zheng et al., 2004).

#### SRSF1/SRSF2

The HPV16 3′ ss SA3358 is the most commonly used splice site in the HPV16 genome. SA3358 generates early mRNAs encoding early proteins, including E6 and E7. The splicing factor serine/arginine-rich (SR) protein SRSF1 has been reported to enhance splicing to 3′ ss SA3358 by interacting with a splicing enhancer (ACCGAAGAA) located downstream of the splice site SA3358. Since SA3358 is used by the majority of the E6 and E7 mRNAs, high levels of SRSF1 function as proto-oncoprotein to upregulate the expression of E6/E7, further resulting in hyperproliferation and, possibly, cancer occurrence (Somberg and Schwartz, 2010). Another SR protein SRSF2 has been shown to be required for E6E7 mRNA production only in cervical cancer-derived cells. The role of SRSF2 is to maintain E6/E7 mRNA stability and inhibit their decay. The knock-down of SRSF2 in cervical tumor cells results in cell apoptosis, suggesting its oncogenic role in cervical cancer progression (McFarlane et al., 2015).

#### HPVs E2 and E6 Proteins

HPV16 early E2 protein and early oncoprotein E6 have been shown to have an inhibitory effect on HPV16 E6/E7 pre-mRNA splicing (Gomes and Espinosa, 2010). HPV16 E2 and E6 directly bind to the intron located in between SD226 and SA409 through their RNA interaction domain in the C-terminus, resulting in the reduction of the E6*I isoform in HPV16-infected cells (Gomes and Espinosa, 2010). This inhibitory effect may also be due to E2 and E6 interfering with several SR proteins, including SRSF9, SRSF6, and SRSF5. Taken together, these findings demonstrate that HPV16 viral proteins E2 and E6 function as splicing regulatory proteins which affect E6/E7 pre-mRNA splicing through their interaction with SR proteins (Bodaghi et al., 2009).

#### CTCF and SF3B1

The CCCTC-binding factor known as CTCF has been firstly identified as a zinc finger DNA-binding transcription factor. Over the past decade, the new tricks of CTCF has been elucidated, especially its new role as a RNA-binding alternative splicing factor (Gomes and Espinosa, 2010; Shukla et al., 2011; Guo et al., 2012; Monahan et al., 2012; Marina et al., 2016). A previous study has shown that CTCF binds to the HPV18 E2-coding region to increase the production of the E6*II isoform. The mutation of CTCF results in the increased production of unspliced E6 mRNA and spliced E6*I mRNA (Paris et al., 2015). It is reasonable to speculate that the increase of E6*II at the expense of unspliced E6 and E6*I mRNA has interrupted the balance of the E6/E7 ratio, causing infected cell apoptosis. However, the mechanism of CTCF in the regulation of E6/E7 expression was not well understood until recently. Researchers have revealed that the inhibitory effect of CTCF is dependent on CTCF and YY-1 loop formation in the HPV18 genome. The downregulation of YY-1 causes an interruption of loop formation and a reversal of epigenetic silencing (Pentland et al., 2018).

The splicing factor 3B1 (SF3B1) was reported to modulate HPV16 E6/E7 pre-mRNA splicing, resulting in increased E6*I in HPV16-positive head and neck cancer cells. The treatment of SF3B1 inhibitor meayamycin B causes the downregulation of the transcript E6*I but promotes the level of the unspliced E6 mRNAs (Gao et al., 2014). These findings further suggest the anti-oncogenic role of SF3B1 in HPV16-derived tumorigenesis. However, the exact mechanism of how SF3B1 affects HPV16 splicing has not been elucidated.

## Conclusion

At present, there is no effective treatment for HPV infection and HPV-related cancer. Although there are vaccines against several high-risk HPV types, the vaccines have no effect on infected patients, and vaccines are not universally accessible due to the high price. Therefore, continuous research on the carcinogenic mechanism of HPV is of great significance for the prevention and treatment of HPV infections and their related cancers. As to the oncogenic proteins expressed in the early replication cycle of high-risk HPVs, the splicing regulation mechanism of E6 and E7 pre-mRNA has a profound impact on the entire replication cycle of HPVs. Moreover, the differentiation, proliferation, and immortalization transformation of HPV-infected cells are tightly related to the level of E6 and E7. Transcripts encoding E6 and E7 proteins are derived from the same pre-mRNA; the selection of splice site SA409 in HPV16 yields the most abundant isoform E6*I, which is further translated into the oncoprotein E7; the unspliced mRNA is translated into the oncoprotein E6 since it contains the complete E6-coding region. Such a mechanism indicates that a perfect balance between E6 and E7needs is required. In HPV16, if the splicing from SA409 becomes too efficient, the production of E6*I will be upregulated, resulting in increased E7 level, but E6 level is decreased, thereby causing apoptosis of the infected cells. Conversely, if the unspliced E6 mRNA is upregulated, the level of E7 will decrease, and the fate of the infected cells will go toward apoptosis. This balancing mechanism between E6 and E7 is regulated by the interaction of cellular splicing factors with cis-regulatory RNA elements on HPV pre-mRNA. A series of RNA regulatory elements and splicing factors are identified to regulate E6/E7 mRNA splicing, indicating that the E6/E7 expression levels are tightly regulated during posttranscription. The role of various splicing factors in HPV-related cancer progression has also been characterized. Overexpression or knockdown of splicing factors will interrupt the ratio of E6/E7 proteins, thereby causing infected cell apoptosis. This weak point of high-risk HPV provides us with an opportunity to identify small molecules that can be used as a splicing factor inhibitor, paving the way to explore more novel antiviral and anticancer drugs.

## Author Contributions

Manuscript writing: YZ and XL. Figure and table making: YJ. Comments and final review: CW. All authors contributed to the article and approved the submitted version.

## Conflict of Interest

The authors declare that the research was conducted in the absence of any commercial or financial relationships that could be construed as a potential conflict of interest.

## Publisher’s Note

All claims expressed in this article are solely those of the authors and do not necessarily represent those of their affiliated organizations, or those of the publisher, the editors and the reviewers. Any product that may be evaluated in this article, or claim that may be made by its manufacturer, is not guaranteed or endorsed by the publisher.
